# Anterior Cervical Hypertrichosis (Hairy Throat Syndrome): Pediatric Case Report and Brief Literature Review

**DOI:** 10.2196/95391

**Published:** 2026-07-14

**Authors:** Nancy Shehata, Husna Irfan Thalib, Heba Alahwal

**Affiliations:** 1 Department of Dermatology, King Abdullah Medical Complex, Prince Nayef Street, Northern Abhor, Jeddah, 23816, Saudi Arabia, 966 504499894; 2 General Medicine Practice Program, Batterjee Medical College, Jeddah, Saudi Arabia

**Keywords:** anterior cervical hypertrichosis, hairy throat syndrome, pediatric dermatology, congenital hypertrichosis, rare skin disorder

## Abstract

Anterior cervical hypertrichosis, also known as hairy throat syndrome, is a rare and typically benign condition characterized by a well-defined patch of terminal hair on the front of the neck. Although it is often an isolated finding, it may sometimes be associated with neurological or developmental abnormalities, which makes clinical awareness important. We report the case of a healthy 4-year-old girl who presented with congenital localized excessive hair growth over the anterior cervical region. The patch had remained unchanged since birth and was not associated with any skin changes or systemic symptoms. Her physical and neurological examinations were normal, and imaging studies ruled out underlying spinal or soft tissue anomalies. Although she had a resolved history of ptosis and was under follow-up for hypermetropia, no other abnormalities were identified. A sibling history of spina bifida prompted a more detailed evaluation, which returned normal results. This case highlights the importance of recognizing anterior cervical hypertrichosis as a rare but distinct clinical entity. Recognition of this benign entity is important to guide appropriate evaluation, avoid excessive investigations, and reduce caregiver anxiety.

## Introduction

Anterior cervical hypertrichosis (ACH), also known as “hairy throat syndrome,” is a rare condition characterized by the presence of a well-defined patch of excessive terminal hair on the anterior aspect of the neck, usually along the midline. It is most often congenital and discovered in early childhood, although some cases may present later [1,2]. The skin underneath the hair typically appears normal, without pigmentation changes, induration, or other cutaneous abnormalities. The condition is generally benign and asymptomatic; however, previous reports have described occasional associations with neurological, skeletal, and ophthalmological abnormalities including peripheral neuropathy, hallux valgus, optic atrophy, and spinal dysraphism [1,3,4]. Therefore, careful clinical assessment and selective investigations may be warranted in some patients depending on the clinical presentation and family history [1-5].

The pathogenesis of ACH is not completely understood but is hypothesized to arise from localized disturbances in hair follicle development during embryogenesis. There is no clear evidence of hereditary transmission in most cases, although rare familial occurrences have been described. Management is generally conservative, focusing on observation and reassurance. In cases where cosmetic concerns are significant, options such as laser hair removal may be considered [3,4].

It is essential to create increased awareness among dermatologists and pediatricians considering its rare occurrence and the potential for misinterpretation or unnecessary investigations. Early recognition and reassurance can prevent parental anxiety and help avoid excessive diagnostic procedures [5].

In this report, we present a case of ACH in a healthy child with no associated systemic abnormalities. We aim to highlight the typical clinical features, discuss the differential diagnoses, and the importance of clinical recognition and parental reassurance in managing this rare condition.

## Case Report

**Figure 1.**
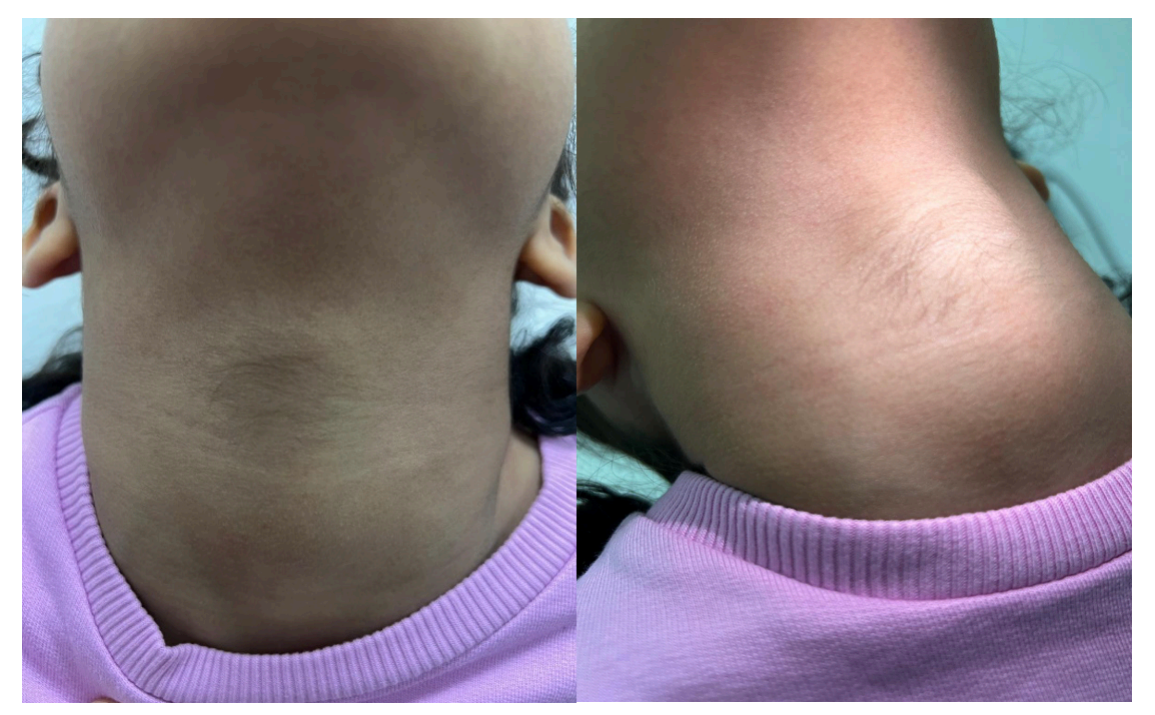
Clinical presentation of anterior cervical hypertrichosis (hairy throat syndrome) with localized excessive hair growth over the anterior neck, showing a well-defined distribution without underlying skin abnormalities.

A 4-year-old medically healthy female child presented to the outpatient clinic with a primary concern of localized excessive hair over the anterior aspect of the neck (Figure 1). The excessive hair growth was noted since birth and has remained unchanged in character or distribution. The excessive hair was localized and presented as a well-defined patch of terminal hair on the anterior neck. There were no associated skin lesions, swelling, pain, or other systemic symptoms. The child did not report any constitutional symptoms such as fever, weight loss, or lethargy. A thorough systemic review was conducted and found to be unremarkable for any neurological complaints.

Upon physical examination, the child appeared well and active. Examination of the neck showed the presence of a well-localized patch of thick terminal hair over the anterior cervical region, with no associated masses, discoloration, or tenderness. Neurological examination did not reveal any signs of central nervous system involvement such as abnormal reflexes, gait disturbance, or limb weakness.

The patient’s past medical history is significant for ptosis, which has since been resolved without the need for surgical intervention. She also has a history of hypermetropia and is currently under regular follow-up with ophthalmology. Her developmental history is normal. There was no history of parental consanguinity. However, family history is notable for a sibling diagnosed with spina bifida, which prompted consideration of possible underlying spinal or neural tube abnormalities in this patient.

Based on the clinical findings and family history, a detailed evaluation plan was initiated. The patient was referred for further assessment by pediatric dermatology consultation. Parents were counseled and educated about the possible causes and implications of hypertrichosis, including rare associations with underlying spinal anomalies such as spinal dysraphism. Considering the reported associations of ACH with skeletal abnormalities and spinal dysraphism, along with the family history of spina bifida, selective investigations were performed. An x-ray of the foot was obtained to evaluate for skeletal abnormalities such as hallux valgus reported in previous cases, while a spinal x-ray was performed as an initial screening tool for occult spinal defects. Neck ultrasound was selected as a noninvasive modality to exclude underlying soft tissue abnormalities. As the patient had no neurological symptoms or abnormal neurological examination findings, advanced imaging such as magnetic resonance imaging was not deemed necessary. All investigations were within normal limits.

## Ethical Considerations

Written informed consent for publication of the patient’s clinical information and photographs was obtained from the patient’s legal guardians. The study was conducted in accordance with institutional ethical standards and the principles of the Declaration of Helsinki. The authors obtained written consent from patients for their photographs and medical information to be published in print and online, and with the understanding that this information may be publicly available. Patient consent forms were not provided to the journal but are retained by the authors.

## Discussion

ACH, also known as “hairy throat,” is a rare form of localized hypertrichosis characterized by the presence of a localized patch of terminal hair on the front of the neck. Approximately 40 cases of ACH have been reported in the literature to date, as summarized in Table 1. While ACH is typically an isolated finding, it can be associated with systemic conditions such as neurological abnormalities (peripheral neuropathy, developmental delay, and mental retardation), ophthalmological disorders (optic atrophy, chorioretinal changes), hallux valgus, and dorsal hypertrichosis. Therefore, it is highly recommended to take a detailed family history and conduct comprehensive clinical examinations and investigations (neurological and ophthalmological assessments, electromyography, and x-ray of the feet) for all patients with ACH to rule out any potential associated abnormalities.

**Table 1.** Literature review of reported cases of anterior cervical hypertrichosis: clinical features and associated findings.

Study	Age and gender	Description of ACH^a^	Neurological	Ophthalmological	Skeletal	Dermatological	Diagnosis	Treatment	Family history
Our case	4-year-old female	Localized excessive hair over anterior neck, present since birth, unchanged in character or distribution	No neurological complaints or findings	Ptosis (resolved), hypermetropia	X-ray of the foot normal (no skeletal anomalies)	Localized hypertrichosis over anterior cervical region	Clinical diagnosis	Referred to pediatric dermatology, parental counseling, investigations normal	Sibling with spina bifida
Chahoub et al [6] (2023)	12-year-old male	Localized patch of hair at the mid-neck region, fine, brown hairs, 3 cm in length	None reported	None reported	None reported	Congenital localized hypertrichosis	Dermoscopy and clinical examination	Laser hair removal (good response)	No family history mentioned
Sawatkar et al [7] (2023)	8-year-old female	Localized terminal coarse hair on anterior neck above the laryngeal prominence	None reported	None reported	None reported	ACH	Sporadic ACH, no consanguinity	None reported	No family history mentioned; sporadic ACH
Cutrone et al [8] (2022)	11-year-old girl and 3-year-old girl	Localized patch of terminal hair on the anterior neck, mostly placed on the hyoid region	None reported	None reported	None reported	None reported	Clinical examination	Laser hair removal	No family history mentioned
Kumar and Das [9] (2021)	8-year-old girl	Localized patch of hair over the laryngeal prominence	None reported	None reported	None reported	None reported	Clinical examination	Laser epilation offered but refused	No family history mentioned
Saini et al [10] (2021)	12-year-old girl	Patch of terminal hairs on the anterior neck above the laryngeal prominence	No	No	No	Yes (nevoid hypermelanosis)	Clinical examination	Laser hair removal	No family history mentioned
Blasco-Morente and Sánchez-Carpintero [5] (2017)	13-year-old female	Terminal hairs in the anterior midline region of the neck	None	None	None	Hypertrichosis	Isolated anterior cervical hypertrichosis	Laser epilation (partial improvement after 3 sessions)	None
Bostan et al [2] (2016)	15-year-old female	Localized patch of terminal hair on the anterior neck above the laryngeal prominence	None reported	None reported	None reported	None reported	Clinical examination, no special diagnostic approach mentioned	Laser hair removal	No family history mentioned
Megna et al (2015) [1]^b^									
Megna et al (2015)	7-year-old Italian girl	Localized patch of terminal hair on the anterior neck	None reported	None reported	None reported	None reported	Clinical examination	Not specified	No history of hypertrichosis or any other skin or hair disease in her family
Meziane et al (2014)	4 patients aged 5‐21 years	Patch of terminal hairs on anterior neck near cricoid cartilage	None reported	None reported	Yes (skeletal abnormalities), chronic juvenile idiopathic arthritis	Yes (hypertrichosis on other parts)	Clinical examination, x-rays, family history, histological examination	Laser hair removal, epilation	No family history mentioned
Reddy and Antaya (2010)	Two unrelated Hispanic females, aged 4 and 3 years	Solitary patch of excessive terminal hair growth in the midline of the neck	None reported	None reported	None reported	None reported	Clinical examination	Not specified	No family history mentioned
Echeverría et al (2010)	56-year-old woman and two daughters (27 and 35 years old, all female)	Congenital localized patch of terminal hair on anterior neck, hyperpigmented plaques with skin thickening on abdominal and lumbar areas	Dysesthesia (in daughter with Down syndrome), overlapping of fourth and third toes (daughter)	None reported	Overlapping toes (daughter with Down syndrome)	Morphea, congenital hypertrichosis	Familial anterior cervical hypertrichosis with systemic associations	Skin biopsy (morphea), liver function tests (chronic liver disease)	Present in 3 family members (maternal side)
Moreno-Giménez et al (2009)	27-year-old female (sporadic ACH case)	Congenital localized patch of terminal hair on anterior neck, no associated abnormalities	None reported	None reported	None reported	None reported	ACH	None reported	Sporadic, nonfamilial ACH in this case
Heitink et al (2007)	13-year-old female	Long, dark blond hair localized in anterior neck	None reported	None reported	None reported	None reported	Clinical examination, x-ray	Intense pulsed light treatment	One sister had spina bifida
Thienpont et al (2006)	No mention found	Anterior cervical hypertrichosis	Yes (mental retardation mildly dysmorphic facial)	Yes (hypermetropia)	Yes (skeletal abnormalities)	Yes (lumbosacral hypertrichosis)	Clinical examination, family history, imaging	Not specified	Family history of similar dysmorphic features and hypertrichosis in multiple affected relatives
Nanda et al (2006)	6 Arab children (aged 9, 3, 1, 12, 10, and 7 years, all female)	Localized hypertrichosis on anterior neck, present since birth or early childhood	None reported	None reported	None reported	Hypertrichosis	Isolated anterior cervical hypertrichosis	No treatment reported	Present in 5 cases (familial)
Corona-Rivera et al (2005)	Mexican boy, age not specified	Anterior cervical hypertrichosis	Yes (mental retardation, EEG^c^ abnormalities, microcephaly)	None reported	Yes (hallux valgus)	Yes (hypertrichosis on back)	Clinical examination, EEG, brain MRI^d^, x-rays	Laser hair removal	No family history mentioned
Hae-Woong Lee et al (2005)	28-year-old female	Increased fine hairs on the anterior cervical area	None reported	Mild myopia	None	Hypertrichosis	Familial anterior cervical hypertrichosis	Epilative laser therapy (refused)	Aunt and cousin also affected; no other abnormalities
Ardinger et al (1992)	13-year-old female and 38-year-old female (mother)	Patch of long hairs on anterior neck above the laryngeal prominence	None reported	None	None	ACH	None	None	None
Trattner et al (1991)	Case 1: 12-year-old male	Patch of long, fair, curly hair in the anterior cervical area	Impaired light touch and temperature perception, complete absence of pain perception in feet, reduced sensory action potentials, motor conduction abnormalities, spina bifida	Bilateral optic atrophy, central scotoma	Bilateral hallux valgus, osteolytic changes, fractures	Anterior cervical hypertrichosis	Osteomyelitis secondary to peripheral sensory neuropathy	Antibiotics (cefotaxime, piperacillin, amikacin, aztreonam, tobramycin, clindamycin), surgical drainage, immobilization	Multiple consanguineous marriages, affected mother and aunt
Trattner et al (1991)	Case 2: (mother) 30-year-old female	Long dark hair patch in the anterior cervical area, present since the first year of life	Decreased pain perception in both legs	Normal	Bilateral hallux valgus, mild kyphoscoliosis	Anterior cervical hypertrichosis	Peripheral sensory and motor neuropathy	No treatment mentioned	First cousin marriage, affected son and sister
Trattner et al (1991)	Case 3: (aunt) 32-year-old female	Localized patch of long, fair hair in the anterior cervical area, present since the first year of life	Subclinical peripheral sensory neuropathy in upper and lower extremities (discovered on EMG)	Normal	Bilateral hallux valgus	Anterior cervical hypertrichosis	Peripheral sensory neuropathy	No treatment mentioned	First cousin marriage, affected nephew and sister

a ACH: anterior cervical hypertrichosis.

b Studies in this category were identified in the review by Mengna et al [1].

c EEG: electroencephalogram.

d MRI: magnetic resonance imaging.

Trattner et al (as reviewed by Megna et al [1]) first reported ACH in three patients from Arab families with consanguineous marriages. All three patients exhibited peripheral neuropathy and bilateral hallux valgus, while one also had bilateral optic nerve atrophy and macular dysfunction. Tsukahara and Kajii [3] later documented ACH in seven members of a Japanese family across three generations with no other associated pathology found, except for one patient with Turner syndrome. ACH has been observed in individuals ranging from birth to early childhood [4], with both familial and sporadic cases reported. The inheritance patterns vary among hereditary cases, typically following an autosomal dominant pattern, though autosomal recessive and X-linked dominant inheritance have also been noted [4]. Additionally, there are sporadic cases with no family history of the condition [2-5].

Our patient represents a sporadic presentation of ACH, as there was no family history of the condition or evidence of associated neurological or skeletal abnormalities on evaluation. Although the patient had a history of resolved ptosis and hypermetropia, no syndromic association was identified. Previous literature reviews have reported peripheral sensory and motor neuropathy as the most commonly associated abnormality, followed by hallux valgus and ophthalmological findings [4-10]. While ACH is primarily a benign cosmetic condition that may cause psychological distress, awareness of its occasionally reported systemic associations remains important. Appropriate clinical assessment, selective investigations guided by history and examination findings, and parental reassurance are essential components of management. Cosmetic treatment options including laser epilation, electrolysis, and intense pulsed light therapy may be considered in selected cases when aesthetic concerns arise [5].

## Abbreviations

ACH: anterior cervical hypertrichosis
